# High-Throughput, Automated Molecular Replacement for Small Molecule MicroED Data

**DOI:** 10.1063/4.0000926

**Published:** 2025-10-27

**Authors:** Adam Thibodeaux, Emma Rova-Danelius

**Affiliations:** University of California Riverside, Riverside, CA, USA

## Abstract

Interest in Microcrystal Electron Diffraction (MicroED) for structural characterization of both proteins and small molecules has dramatically risen since the method’s inception 12 years ago. While *ab initio* phasing methods remain the gold standard for small molecule MicroED data, radiation beam damage during data collection and poor crystallinity of the sample makes this method unfeasible in many cases, commonly in the case of complex molecules with some degree of inherent flexibility and large substituents. Molecular replacement (MR) is a very common phasing method for protein MicroED data that can circumnavigate this diminished data quality, however MR has seen little traction with small molecules due to the dearth of diverse methods to efficiently sample the conformational landscape of small molecules. Herein, a method based on high- throughput, automated molecular replacement has been developed to rectify this issue. This method was used to solve three macrocycle structures with known MicroED structures up to 2.0Å resolution and one novel structure at 0.97Å resolution that was unable to be solved with *ab initio methods*. This method may find uses for larger and more complex molecules including large peptides, toxins and natural products, which typically generates diffraction data with resolutions >1.0 Å.

